# The Tubercular Patients Problem

**Published:** 1916-01-08

**Authors:** 


### THE TUBERCULAR PATIENTS PROBLEM.

Signs are not wanting which point to a more drastic selection of cases recommended for sanatorium treatment. In London the financial position is to be met by reducing the number of beds reserved for insured consumptives, and this will mean that only those cases who are likely to be more or less permanently benefited will be admitted to institutions for treatment. This will not diminish the difficulties of those workers, medical and lay, who are engaged in fighting tuberculosis. The insured person who finds himself suffering from consumption is the last person to view with scepticism the claims made for sanatorium treatment. He has paid his contributions, and is inclined to demand a place in one of "the first-class hotels."

The solution of the tuberculosis problem is thus hampered by the claims of the tuberculous case. It is manifest that some of the expense incurred in the treatment of consumptives not only fails to assist in diminishing the incidence of tuberculosis, but, on the contrary, actually tends to propagate the disease by prolonging the infectious period of the patient's life, and thereby his capacity for infecting others. The number of cases sent to sanatoria who can, at the best, be only patched up temporarily is unfortunately a high one. In six months or a year these patients are again incapacitated and more infectious than ever. Then it is that the medical officer of health and the tuberculosis officer deplore the lack of adequate segregation accommodation. "Hospital" beds, in contradistinction to "sanatorium" beds, are as yet too few, and the patient is confronted with the alternatives of being a dangerous burden at home or of accepting a bed in a Poor-Law infirmary; the special tuberculosis wards in which, however much they have been improved, do not appeal to the insured consumptive, as they lack the least connection with sanatorium benefit, towards which his compulsory contributions were supposed to be directed.

It has been stoutly maintained by competent authorities that the cheapest and most productive measure in the fight against consumption is that directed towards the segregation of the advanced and infectious sufferers. By this means the supply of bacilli is cut off to a very large extent, and the enemy is deprived of ammunition. To do this on a large scale is perhaps beyond the range of practical politics of the moment, but it is gratifying to note an increasing tendency in this direction.

But there is one other direction in which money may be spent without fear of criticism on the score of doubtful utility—namely, in the extension of the principle of the open-air school. It is not necessary that a diagnosis of tubercle should be made in the case of any particular child before it is considered eligible for such a school. It is, indeed, notoriously difficult to assert the presence or absence of tuberculosis in school-children; but the whole point of the existence of the open-air school lies in the fact that the vast majority of children sent there will substantially benefit. The frail, the badly nourished, or weedy child will have his or her resistance raised, not only against tubercle, but against other diseases. Whether the seeds of consumption are present or not, the cost of open-air schools or children's sanatoria is bound to be repaid in adolescence, for much of the adult tuberculosis is simply a late manifestation of infection in childhood or re-infection in early youth. The profitableness of expenditure in this direction seems to be unquestionable. In reviewing, then, the problems of the anti-tuberculosis campaign it appears that the sanest and safest procedures are those having for their objective the segregation of the advanced cases and the extensive application of the principle of the open-air schools to all delicate or "pre-tubercular" children.

